# Preamplification Procedure for the Analysis of Ancient DNA Samples

**DOI:** 10.1155/2013/734676

**Published:** 2013-09-25

**Authors:** Stefania Del Gaudio, Alessandra Cirillo, Giovanni Di Bernardo, Umberto Galderisi, Theodoros Thanassoulas, Theodoros Pitsios, Marilena Cipollaro

**Affiliations:** ^1^Department of Experimental Medicine, Section of Biotechnology and Molecular Biology “A. Cascino”, Second University of Naples, Via Costantinopoli 16, 80138 Naples, Italy; ^2^Department of Cardiothoracic Sciences, Second University of Naples, Via Leonardo Bianchi, 80131 Naples, Italy; ^3^Anthropology Museum of Athens Medical School, Medical School, 75 M. Asias Street 11527 Goudi, Greece

## Abstract

In ancient DNA studies the low amount of endogenous DNA represents a limiting factor that often hampers the result achievement. In this study we extracted the DNA from nine human skeletal remains of different ages found in the Byzantine cemetery of Abdera Halkidiki and in the medieval cemetery of St. Spiridion in Rhodes (Greece). Real-time quantitative polymerase chain reaction (qPCR) was used to detect in the extracts the presence of PCR inhibitors and to estimate the DNA content. As mitochondrial DNA was detected in all samples, amplification of nuclear targets, as amelogenin and the polymorphism M470V of the transmembrane conductance regulator gene, yielded positive results in one case only. In an effort to improve amplification success, we applied, for the first time in ancient DNA, a preamplification strategy based on TaqMan PreAmp Master Mix. A comparison between results obtained from nonpreamplified and preamplified samples is reported. Our data, even if preliminary, show that the TaqMan PreAmp procedure may improve the sensitivity of qPCR analysis.

## 1. Introduction

The low amount of endogenous DNA molecules is one of the limiting factors in ancient DNA (aDNA) studies [1–5], together with the presence of PCR inhibitors, the degradation and damage of endogenous DNA, and the risk of exogenous DNA contamination.

A variety of DNA extraction and purification techniques have been developed with the aim to retrieve as much DNA as possible, while minimizing the coextraction of PCR inhibitors [6–8]. In particular, the presence of inhibitors introduces a number of issues, ranging from the reduced amplification efficiency and assay sensitivity to the complete amplification failure. Dilution of the template DNA and consequently of the inhibitors and/or the addition of PCR facilitators might circumvent this problem [9, 10].

 DNA extracted from ancient samples is often fragmented down to 100–300 bp or less [11], and thus the probability of a successful amplification increases when short amplicons are chosen to be amplified. Moreover, several biochemical modifications, other than fragmentation, occur typically in ancient DNA samples, causing unextendable DNA strand or base misincorporations [12, 13]. Enzymatic repair strategies have been proposed in order to restore DNA molecules [14] and consequently enhance the amount of amplifiable molecules, thus increasing the success rate in analysing nuclear genes in animal [15] and human remains [16]. 

Since ancient samples contain low copy number and/or highly degraded DNA, multicopy mitochondrial DNA (mtDNA) is a good candidate to be analyzed. Nonetheless, mtDNA testing is characterised by a lower informative power than nuclear DNA [17]. Unfortunately, the low copy number of nuclear loci is a strong limitation to the PCR amplification. To overcome this limitation, different preamplification approaches have been proposed, including the degenerated oligonucleotide-PCR (DOP-PCR) [15, 18] and primer extension-PCR [19] both aiming to an enhanced amplification of ancient DNA molecules.

This study is focused on circumventing the low amount of target DNA molecules through the use, for the first time in the ancient DNA field, of the TaqMan PreAmp Master Mix, which enables the preamplification of DNA with pooled primers targeting different loci, followed by specific real-time quantitative PCR (qPCR) amplifications [20, 21]. We previously demonstrated that this procedure allows a linear amplification and enhances the sensitivity and the precision of qPCR analysis of food matrices where DNA molecules are present in low amounts and/or in degraded forms [20].

In the present work we evaluated the mitochondrial content of DNA samples retrieved from nine human skeletal remains of different ages (2nd century B.C.–16th century A.D.), coming from the Byzantine cemetery of Abdera Halkidiki and the medieval cemetery of St. Spiridion in Rhodes (Greece), by a qPCR amplification of a 92 bp fragment of the mitochondrial control region [22]. Moreover we carried out qPCR assays specific for a segment of the X-Y chromosome homologous amelogenin (AMG) gene [2, 22] to achieve both nuclear DNA quantification and sex determination and for the transmembrane conductance regulator (CFTR) gene polymorphism M470V [23], selected as a representative nuclear locus. 

DNA extracts were investigated either by direct qPCR amplification and by qPCR of each target preceded by a preamplification step, allowing a comparison between results obtained from nonpreamplified and preamplified DNA samples.

The AMG preamplification assay was also tested on previously characterized skeletal remains from a Pompeii archaeological site [24, 25].

## 2. Materials and Methods

### 2.1. Working Conditions

Ancient human remains were treated under the following conditions: DNA extraction and pre-PCR and PCR steps were carried out in physically separated laboratories, all surfaces and instruments were bleached, and plasticware and solutions underwent UV irradiation. The operators wore fresh body protection for each pre-PCR step and wore gloves and face masks throughout the entire pre-PCR and PCR work. Disposable, gamma-irradiated pipette tips (Diamond; Gilson Middleton, WI, USA), sterile 1.5 and 2.0 mL microcentrifuge tubes (Biopur; Eppendorf, Hamburg, Germany), and gamma-irradiated 0.2 mL PCR tubes (Molecular BioProducts Inc., San Diego, CA, USA) were used. Modern DNA extraction and handling occurred in a separate laboratory by an operator that did not participate in ancient DNA analysis.

### 2.2. Bone Material and Samples Pretreatment

The bone samples used for this study were obtained from nine ancient skeletons of different ages (2nd century B.C.–16th century A.D.) found in the Byzantine cemetery of Abdera Halkidiki and in the medieval cemetery of St. Spiridion in Rhodes (Greece) and collected, at present, in the Museum of Anthropology of the University of Athens. As control, we analysed two bone remains belonging to skeletons found in Caius Iulius Polybius house in Pompeii (79 A.D.), whose molecular data were previously reported [24, 25]. Bone samples were treated to avoid contamination as already described [25].

### 2.3. Ancient DNA Extraction

DNA was extracted from ancient samples using a previously reported procedure [25, 26] with slight modifications. One hundred milligrams of bone powder was demineralized in 1.5 mL of EDTA 0.5 M (pH 8.0) and 100 *μ*L of 20 mg/mL proteinase K in a rotary shaker. After incubation at 56°C overnight, 500 *μ*L of lysis solution (4 M GuSCN, 0.1 M Tris-HCl pH 5.4, 0.02 M EDTA pH 8.0, and Triton X-100 1.3%) was added, and the mixture was incubated at 56°C overnight. We introduced an incubation step with a phenyl-thiacyl-thiazolium-bromide solution (PTB) [27, 28] which was followed by heating at 94°C for 10 min. After centrifugation, the supernatant was separated in two aliquots that were incubated with 1 volume of binding buffer containing 6 M sodium iodide and 25 *μ*L of silica suspension [29]. In agreement with [9], the pH of this mixture was adjusted to ~4.0 with hydrochloric acid. After incubation in ice for 2-3 h, the silica pellet was washed twice with washing buffer (0.01 M Tris-HCl pH 7.5, 0.05 M NaCl, and 1 mM EDTA pH 8.0), twice with absolute ethanol, and twice with 70% ethanol and air-dried for 30 min. Finally the DNA was eluted at 56°C for 30 min in 100 *μ*L of sterile distilled water and stored at −20°C. 

### 2.4. Modern DNA Extraction

DNA to be used as Internal Positive Control (IPC) in the inhibition assay was extracted from soy (*Glycine max*) powdered certified reference material (CRM) (Sigma-Aldrich, Seelze, Germany) according to [20, 30]. Extraction of human genomic DNA to be used for the construction of the AMG-X calibration curve was performed from a mesenchymal cell culture with MasterPure DNA Purification Kit (Epicentre, Madison, WI). DNA concentrations were measured using the Nanodrop ND-1000 spectrophotometer (Thermo Scientific, Wilmington, DE).

### 2.5. Primers and Probes

Primers and TaqMan probe sequences specific for soy lectin gene were as already reported [20]. We designed a TaqMan minor groove binder (MGB) assay specific for a 92 bp fragment of the coding region of the human mitochondrial genome, from nucleotides 8345 to 8436, with the aid of Primer Express 5.0 software (Life Technologies, Forster City, CA), modifying the upper primer and the probe sequences used elsewhere [22]. As a standard for mitochondrial quantification, a 360 bp region, from nucleotides 8200 to 8559, containing the 92 bp fragment was amplified. The qPCR specific for the X-Y homologous amelogenin (AMG) gene was performed as already described [2]. MGB probes were purchased from Life Technologies while all the primers and the soy-specific TaqMan probe were supplied by Eurofins MWG Operon (Ebersberg, Germany). Primer and probe sequences are listed in Table 1. For the CFTR M470V (1540 A/G) polymorphism detection, we purchased a TaqMan SNP Genotyping Assay from Life Technologies (AB assay ID: C_–––_3021372_-_10).

### 2.6. Inhibition Assay

In order to highlight a potential PCR inhibition in the samples which we analysed, we evaluated the effect of each ancient DNA extract on the PCR amplification of the soy IPC [31]. We selected the lectin as target gene, a taxon-specific DNA sequence of the soy. The real-time TaqMan quantitative assay [20, 32] included a standard reaction (the IPC only) other than a sample reaction (the IPC and the aDNA extract each representing the 10% of the final PCR volume), each performed in triplicate on a DNA Engine Opticon 2 MJ research (Biorad, Hercules, CA). Two *μ*L of DNA was amplified in a total volume of 25 *μ*L containing 1X TaqMan Universal Master Mix (Life Technologies), and the thermal protocol was as follows: 2 min at 50°C for the activation of the uracil-DNA-glycosylase, 10 min of denaturation at 95°C followed by 50 cycles of a two-step program (denaturation at 95°C for 15 sec and annealing/extension at 60°C for 1 min). Threshold cycle (Ct) values of each reaction were measured by Opticon Monitor 2 software, and Ct shifts (ΔCt) were calculated as the difference between the average Ct of the sample and of the Ct of the standard reactions. The expected recovery (ER) value was calculated according to the formula

(1) $$\begin{array}{c} {\left(\text{dilution}\;\text{factor}\times 2^{\Delta \text{Ct}}\right)}^{-1}\times 100\%. \end{array}$$

### 2.7. PreAmp Procedure

Primers targeting mitochondrial coding region and AMG gene, listed in Table 1, were pooled to prepare a mix containing 9 pmol of each primer together with 1X TaqMan SNP Genotyping Assay. The reaction conditions are based on the amplification of 10 *μ*L of aDNA in a 25 *μ*L reaction mix containing 12.5 *μ*L of TaqMan PreAmp Master Mix (Life Technologies, Foster City, CA) and 1.15 *μ*L of pooled primers mix. A no-template control was included with each experiment. PreAmp reaction was carried out on a Veriti Thermocycler (Life Technologies) with the following program: denaturation step at 95°C for 10 min followed by 14 cycles of amplification (15 sec at 95°C, 4 min at 60°C). Two *μ*L of the preamplified product was used undiluted for the subsequent real-time PCR assay.

### 2.8. qPCR

The qPCR reactions for mitochondrial coding region and nuclear genes (AMG and CFTR polymorphism M470V) were performed in triplicate on a DNA Engine Opticon 2 MJ research (Biorad, Hercules, CA). PCR experimental conditions were as described for the inhibition assay. Each PCR included two negative controls, that is, a blank PCR and a blank extraction control. Moreover, the no-template control was amplified to check for contamination during the preamplification procedure. The mitochondrial calibration curve was constructed through the dilution of the 360 bp standard fragment, to contain 22 × 10^7^ to 22 molecules. For the AMG-X copy number estimation, we included twofold serial dilutions of the female genomic DNA ranging from 16000 pg (5533 copies) to 130 pg (43 copies), assuming 3 pg as the haploid human genome content of a single cell. Ct values were plotted against log_10_ of the copy number to generate standard curves. The copy number value for aDNA samples was inferred from the regression line of the standard curves. Standard deviations (SD) and coefficients of variation (CV) were evaluated for triplicate measurements. 

### 2.9. Statistical Analysis

Statistical analysis was performed using GraphPad software (Prism 5). The two-way analysis of variance (ANOVA) was applied to the experimental data sets derived from preamplified and nonpreamplified sample groups followed by Bonferroni's multiple comparison tests. A probability value of *P* < 0.05 was considered statistically significant.

## 3. Results and Discussion

Quantification of mitochondrial and nuclear DNA has become a crucial analysis in ancient DNA research [5, 33, 34]. The estimation of the number of template DNA molecules is useful to ensure the reliability of PCR-based studies on low copy number and/or highly damaged DNA samples [2, 22]. A negative PCR result can be due, other than to the low copy number of DNA molecules, also to the presence of Taq DNA polymerase inhibitor compounds in the DNA extract [33]. Several authors recommend testing extracts for inhibition, in order to optimize sample dilution and to maximize accuracy of quantitative analysis and/or template recovery of subsequent PCR [31, 35]. Accordingly, we used qPCR to detect the presence of inhibitors by analysing the Ct deviation of a spiked IPC. We chose as IPC a DNA extracted from soy flour for the following reasons:

- soy DNA was a nonendogenous template;
- since our laboratory is accredited for qualitative and quantitative testing of genetically modified organisms (GMO) in food according to EN ISO/IEC 17025: 2005, we have to use validated qPCR protocols. This is the case of the lectin-specific assay used for the inhibition test for which we previously carried out a performance study [20].

We amplified the IPC alone and the IPC spiked with each ancient sample. Inhibition was detected on the basis of the ΔCt of the spiked positive control. The ER value was assumed as an estimate of the effect of inhibition and of the extract dilution on subsequent amplifications of different gene targets from the same sample [31]. All aDNA extracts showed a complete inhibition when tested undiluted indicating that the inhibitors were not completely removed during extraction step. A 1/10 dilution allowed the amplification of the IPC with ΔCt values ranging from 0.4 to 0 but resulting in a very low ER value (between 7.1% and 10%), meaning that we expect to recover less than 10% of the template available in the original extracts. On the basis of these results, we performed the subsequent PCR amplifications using 1/10 dilutions of the aDNA extracts.

For the mtDNA quantitative analysis we selected a target encompassing the tRNA lysine and the ATP synthase 8 genes in the coding region of the mitochondrial genome, where a few rare polymorphic sites were reported [23]. As shown in Table 2, the mtDNA was detected in all the samples, with a very low starting copy number (~10 copies/*μ*L), except for the A-1V sample, containing about 930 copies/*μ*L. This result agrees with the bone type and its preservation conditions, since A-1V was the best preserved sample. Nuclear DNA quantification was performed by a MGB duplex real-time assay specific for the X-Y homologous amelogenin gene that allowed the simultaneous estimation of the AMG-X 106 bp fragment (FAM-labeled) and of the AMG-Y 112 bp fragment (VIC-labeled), thus making possible not only the DNA quantification but also the sex determination [2, 22]. Quantitative analysis of the AMG-X allele, shown in Table 2, yielded negative results for all samples except for A-1V (250 copies/*μ*L). The AMG-Y assay was used as qualitative assay to confirm the sex determination. No detectable fluorescence signal was observed in any of the PCR or extraction blanks.

For what concerns the qualitative analysis, we were able to determine the sex of A-1V sample only, which was a female, while no data were obtained for all other samples. Analysis of the M470V polymorphism of CFTR gene yielded negative results in all the samples. This is not surprising since it is known that low copy number (less than 100 mtDNA molecules/*μ*L) samples offer more difficulties to obtain high quality results for nuclear targets as reported by [2, 5, 34] for DNA sequencing and by [1, 3, 36, 37] for microsatellite typing.

The first strategy we applied to improve the sensitivity of AMG and CFTR typing was based on amplification replicates for each extract with different DNA inputs (taking into account that increased DNA amount is related to the inhibition susceptibility of the PCR analysis), but this approach failed to work. In order to enhance the amplification sensitivity, we decided to develop a strategy based on the preamplification [20, 21] of mitochondrial and nuclear DNA followed by specific single-target real-time PCR assays. 

The protocol of preamplification we used was as follows:

- the forward and reverse primers targeting the genes of interest (mtDNA coding region, AMG, and CFTR genes) were pooled;
- the pool was combined with the TaqMan Preamp Master Mix (Life Technologies) and the aDNA extract to obtain a reaction mixture that underwent 10 or 14 cycles of preamplification;
- the preamplified product was used as template for a qPCR assay specific for each target gene.

We performed several experiments to optimize the PreAmp conditions, varying the quantity of the DNA extract, the primer concentration in the pooled mix, the cycling conditions, and the dilution factor of preamplified DNA (data not shown). We obtained the best performance using the reaction conditions reported in Section 2.7.

The preamplification strategy allowed an increase in mitochondrial copy number of at least 1000-fold for all the samples that we analysed (Table 2). Furhermore, we were able to increase the AMG copy number in three extra samples in addition to A-1V sample. As far as the biological sex determination (Table 2), preamplification allowed us to confirm that the A-1V sample was a female and to recover information also for B2, B5, B7, and A-2 samples. For what concerns the CFTR typing, we obtained data for samples B2, B5, B7, B9, and A-1V. To avoid mistyping, all samples were analysed a minimum of three times. The no-template control was always negative.

Table 3 shows the mean value, the SD, and the CV of Ct values obtained in amplifying the mitochondrial and AMG-X targets. Preamplified samples, as compared to nonpreamplified DNA, showed an extremely significant improvement of the Ct value (*P* < 0.0001), ranging from 10.0 to 14.4 for mitochondrial target, suggesting that preamplification step enhances the sensitivity of mitochondrial real-time PCR assay. The lack of quantitative data on AMG-X allele did not allow the confirmation of this concept also for nuclear targets.

The precision of a quantitative method is expressed in terms of CV value, which is the ratio of the standard deviation of the mean divided by the mean value of measurements. As far as the precision of the mitochondrial quantitative assay in this study, a nonsignificant decrease of CV of Ct mean in the majority of samples (7/9) was observed (Table 3). Additional experiments are required to address the improvement of this parameter in quantitative analysis of ancient DNA samples.

Another advantage of this strategy is the possibility to increase the number of targets that can be analyzed from each preamplified extract compared with the direct qPCR.

 Other authors demonstrated that the preamplification technique does not seem to produce false results [20, 21]. In this study we have only an indication of the results reliability since in the A-1V sample the AMG-X alleles detection by direct qPCR is confirmed in the preamplified DNA. The A-2 sample was classified as a male, although the X signal was absent, since we were able to detect the Y allele. We hypothesize that the extensive DNA degradation in ancient human bone samples has played a key role in the amplification failure since it decreased the number of amplifiable molecules up to a high allelic dropout incidence [2]. 

Unfortunately, no anthrophological data on sex determination were available to confirm our results on Greek remains object of this study. DNA extracted from two skeletal remains of Pompeii archaeological site were preamplified and then submitted to the amplification assay for AMG. For these samples, our research group already reported histological and anthropometric evaluation along with successful amplification of single copy gene useful for biological sex determination, AMG and Y specific alphoid repeats [24, 25]. We compared the new data with those already obtained with the classical amplification protocol [24, 25], confirming that 1A and 1B skeletons belonged to a male.

## 4. Conclusions

 Our data show that the TaqMan PreAmp procedure enhances the sensitivity of quantitative analysis as a significant improvement of Ct values of mitochondrial target was found comparing nonpreamplified versus preamplified DNA samples. These results, even if preliminary, support utility of the preamplification in order to recover genetic data from degraded samples. More work needs to be done concerning the investigation of samples of known DNA profile (such as STR), to evaluate the sensitivity of the procedure and to exclude possible PCR amplification artifacts. As suggested elsewhere [20, 21] this strategy will be successfully applicable to other fields that require qualitative and quantitative testing by qPCR of low copy number and highly degraded DNA samples.

## Figures and Tables

**Table 1:** List of primers and probes. For each target name, sequence, concentration used, amplicon size, and reference are shown.

Target	Primer and probe names	5′ Sequence	Concentration (nM)	Amplicon size (bp)	Ref.
Soy lectin	GM1-F GM1-R Probe GM1	CCAGCTTCGCCGCTTCCTTC GAAGGCAAGCCCATCTGCAAGCC FAM-CTTCACCTTCTATGCCCCTGACAC-TAMRA	600 600 120	74	[20, 32]

Human mtDNA	mt coding-F mt coding-R Probe coding	CCAACACCTCTTTACAGTGAAATGC GTGATGAGGAATAGTGTAAGGAGTATGG FAM-CAACTAAATACTACCGTATGGC-MGB	300 300 200	92	[22] This work

Human mtDNA standard	mt cod 8200F mt cod 8559R	GTTTCATGCCCATCGTCCTA GCAATGAATGAAGCGAACAG	100 100	360	This work

Human AMG X/Y	AMG-F AMG-R Probe AMG-X Probe AMG-Y	CCCTGGGCTCTGTAAAGAATAGTG ATCAGAGCTTAAACTGGGAAGCTG FAM-TATCCCAGATGTTTCTC-MGB VIC-CATCCCAAATAAAGTG-MGB	100 100 150 150	106/112	[2]

**Table 2:** Summary of quantitative and qualitative analysis. Mitochondrial and nuclear DNA copy number and AMG and CFTR typing data of nonpreamplified and preamplified DNA extracted from the nine bone samples.

Sample	Bone type	Age	mtDNA (copies/*μ*L)		AMG X (copies/*μ*L)		AMG X/Y typing		CFTR typing	
			Nonpreamplified	Preamplified	Nonpreamplified	Preamplified	Nonpreamplified	Preamplified	Nonpreamplified	Preamplified
B1	Carpal bone	2nd century B.C.– 8th century A.D	<10	1.2 × 10^4^	—	—	—	—	—	—
B2	Carpal bone		<10	7.6 × 10^5^	—	5.8 × 10^5^	—	XX	—	AG
B4	Carpal bone		10	1.1 × 10^4^	—	—	—	—	—	—
B5	Long bone fragment		<10	8 × 10^3^	—	1.4 × 10^3^	—	XX	—	AA
B7	Cranial bone fregment		13	6.3 × 10^4^	—	6.7 × 10^4^	—	XY	—	AA
B9	Cranial bone fregment		<10	2 × 10^3^	—	—	—	—	—	GG

A-1V	Vertebra	13–16th century A.D	9.3 × 10^2^	3 × 10^6^	2.5 × 10^2^	3.4 × 10^5^	XX	XX	—	AG
A-2	Cranial bone fregment		<10	6 × 10^3^	—	—	—	Y	—	—
A-3	Cranial bone fregment		<10	1 × 10^3^	—	—	—	—	—	—

**Table 3:** qPCR analysis of nonpreamplified and preamplified samples. Ct averages, standard deviations (SD), and coefficients of variation (CV) of mtDNA and AMG-X targets from nonpreamplified and preamplified aDNA samples.

Sample	mtDNA Ct (average ± SD) CV		AMG-X Ct (average ± SD) CV	
	Nonpreamplified	Preamplified	Nonpreamplified	Preamplified
B1	37.00 ± 0.28; 0.76%	22.6 ± 0.28 1.25%	—	—
B2	37.35 ± 0.64 1.70%	15.71 ± 0.01 —	—	23.62 ± 0.25 1.05%
B4	34.45 ± 1.06; 3.08%	22.75± 0.07 0.31%	—	—
B5	36.25 ± 0.42 1.16%	23.40 ± 0.71 3.03%	—	25.92 ± 0.05 0.19%
B7	34.05 ± 0.28 0.82%	19.85 ± 0.07 0.35%	—	27.56 ± 0.30 1.11%
B9	37.5 ± 0.42 1.13%	25.35 ± 0.07 0.28%	—	—
A-1V	26.91 ± 0.31 1.15%	13.19 ± 0.18 1.36%	35.77 ± 0.09 0.27%	24.4 ± 0.25 1.06%
A-2	35.70 ± 0.14 0.39%	23.85 ± 0.07 0.29%	—	—
A-3	36.40 ± 0.28 0.77%	26.40 ± 0.01 —	—	—

## References

[1] Alonso A, Andelinović S, Martín P (2001). DNA typing from skeletal remains: evaluation of multiplex and megaplex STR systems on DNA isolated from bone and teeth samples. *Croatian Medical Journal*.

[2] Alonso A, Martín P, Albarrán C (2004). Real-time PCR designs to estimate nuclear and mitochondrial DNA copy number in forensic and ancient DNA studies. *Forensic Science International*.

[3] Gill P (2001). Application of low copy number DNA profiling. *Croatian Medical Journal*.

[4] Anderung C, Persson P, Bouwman A, Elburg R, Götherström A (2008). Fishing for ancient DNA. *Forensic Science International*.

[5] Hofreiter M, Serre D, Poinar HN, Kuch M, Pääbo S (2001). Ancient DNA. *Nature Reviews Genetics*.

[6] Loreille OM, Diegoli TM, Irwin JA, Coble MD, Parsons TJ (2007). High efficiency DNA extraction from bone by total demineralization. *Forensic Science International*.

[7] Rohland N, Hofreiter M (2007). Ancient DNA extraction from bones and teeth. *Nature protocols*.

[8] Rohland N, Siedel H, Hofreiter M (2010). A rapid column-based ancient DNA extraction method for increased sample throughput. *Molecular Ecology Resources*.

[9] Rohland N, Hofreiter M (2007). Comparison and optimization of ancient DNA extraction. *BioTechniques*.

[10] Wilson IG (1997). Inhibition and facilitation of nucleic acid amplification. *Applied and Environmental Microbiology*.

[11] Paabo S (1989). Ancient DNA: extraction, characterization, molecular cloning, and enzymatic amplification. *Proceedings of the National Academy of Sciences of the United States of America*.

[12] Hoss M, Jaruga P, Zastawny TH, Dizdaroglu M, Paabo S (1996). DNA damage and DNA sequence retrieval from ancient tissues. *Nucleic Acids Research*.

[13] Capelli C, Tschentscher F, Pascali VL (2003). “Ancient” protocols for the crime scene? Similarities and differences between forensic genetics and ancient DNA analysis. *Forensic Science International*.

[14] Pusch CM, Giddings I, Scholz M (1998). Repair of degraded duplex DNA from prehistoric samples using Escherichia coli DNA polymerase I and T4 DNA ligase. *Nucleic Acids Research*.

[15] Di Bernardo G, Del Gaudio S, Cammarota M, Galderisi U, Cascino A, Cipollaro M (2002). Enzymatic repair of selected cross-linked homoduplex molecules enhances nuclear gene rescue from Pompeii and Herculaneum remains. *Nucleic acids research*.

[16] Kovatsi L, Nikou D, Triantaphyllou S, Njau SN, Voutsaki S, Kouidou S (2009). DNA repair enables sex identification in genetic material from human teeth. *Hippokratia*.

[17] Vallone PM, Just RS, Coble MD, Butler JM, Parsons TJ (2004). A multiplex allele-specific primer extension assay for forensically informative SNPs distributed throughout the mitochondrial genome. *International Journal of Legal Medicine*.

[18] Pusch CM, Nicholson GJ, Bachmann L, Scholz M (2000). Degenerate oligonucleotide-primed preamplification of ancient DNA allows the retrieval of authentic DNA sequences. *Analytical Biochemistry*.

[19] Satoh K, Takahashi KI, Itoh Y, Kobayashi R (1998). Typing of DNA using the primer extension preamplification (PEP) method—studies of reliability of typing and detection limits-. *Japanese Journal of Legal Medicine*.

[20] Del Gaudio S, Cirillo A, Di Bernardo G, Galderisi U, Cipollaro M (2010). A preamplification approach to GMO detection in processed foods. *Analytical and Bioanalytical Chemistry*.

[21] Noutsias M, Rohde M, Block A (2008). Preamplification techniques for real-time RT-PCR analyses of endomyocardial biopsies. *BMC Molecular Biology*.

[22] Andréasson H, Gyllensten U, Allen M (2002). Real-time DNA quantification of nuclear and mitochondrial DNA in forensic analysis. *BioTechniques*.

[23] Dequeker E, Stuhrmann M, Morris MA (2009). Best practice guidelines for molecular genetic diagnosis of cystic fibrosis and CFTR-related disorders—updated European recommendations. *European Journal of Human Genetics*.

[24] Cipollaro M, Di Bernardo G, Galano G (1998). Ancient DNA in human bone remains from Pompeii archaeological site. *Biochemical and Biophysical Research Communications*.

[25] Di Bernardo G, Del Gaudio S, Galderisi U, Cascino A, Cipollaro M (2009). Ancient DNA and family relationships in a pompeian house. *Annals of Human Genetics*.

[26] Hoss M, Paabo S (1993). DNA extraction from Pleistocene bones by a silica-based purification method. *Nucleic Acids Research*.

[27] Poinar HN, Hofreiter M, Spaulding WG (1998). Molecular coproscopy: dung and diet of the extinct ground sloth Nothrotheriops shastensis. *Science*.

[28] Vasan S, Zhang X, Zhang X (1996). An agent cleaving glucose-derived protein crosslinks in vitro and in vivo. *Nature*.

[29] Boom R, Sol CJA, Salimans MMM, Jansen CL, Wertheim-Van Dillen PME, Van Der Noordaa J (1990). Rapid and simple method for purification of nucleic acids. *Journal of Clinical Microbiology*.

[30] Foodstuffs—methods of analysis for the detection of genetically modified organisms and derived products—nucleic acid extraction.

[31] King CE, Debruyne R, Kuch M, Schwarz C, Poinar HN (2009). A quantitative approach to detect and overcome PCR inhibition in ancient DNA extracts. *BioTechniques*.

[32] Foodstuffs—methods of analysis for the detection of genetically modified organisms and derived products—quantitative nucleic acid based methods.

[33] Alonso A, Martín P, Albarrán C (2003). Specific quantification of human genomes from low copy number DNA samples in forensic and ancient DNA studies. *Croatian Medical Journal*.

[34] Handt O, Krings M, Ward RH, Pääbo S (1996). The retrieval of ancient human DNA sequences. *American Journal of Human Genetics*.

[35] Ramakers C, Ruijter JM, Lekanne Deprez RH, Moorman AFM (2003). Assumption-free analysis of quantitative real-time polymerase chain reaction (PCR) data. *Neuroscience Letters*.

[36] Morin PA, Chambers KE, Boesch C, Vigilant L (2001). Quantitative polymerase chain reaction analysis of DNA from noninvasive samples for accurate microsatellite genotyping of wild chimpanzees (Pan troglodytes verus). *Molecular Ecology*.

[37] Gill P, Whitaker J, Flaxman C, Brown N, Buckleton J (2000). An investigation of the rigor of interpretation rules for STRs derived from less than 100 pg of DNA. *Forensic Science International*.

